# Bioprospecting for Antithrombotic Polar Lipids from Salmon, Herring, and Boarfish By-Products

**DOI:** 10.3390/foods8090416

**Published:** 2019-09-15

**Authors:** Alexandros Tsoupras, Eoin O’Keeffe, Ronan Lordan, Shane Redfern, Ioannis Zabetakis

**Affiliations:** 1 Department of Biological Sciences, University of Limerick, V94 T9PX Limerick, Ireland; Eoin.OKeeffe@ul.ie (E.O.); Ronan.Lordan@ul.ie (R.L.); Shane.Redfern@ul.ie (S.R.); Ioannis.Zabetakis@ul.ie (I.Z.); 2 Health Research Institute (HRI), University of Limerick, V94 T9PX Limerick, Ireland

**Keywords:** platelet aggregation, PAF, thrombin, collagen, ADP, fish, marine, cardiovascular diseases (CVD), PUFA

## Abstract

Marine polar lipids (PLs) have exhibited promising cardioprotection. In this study, marine by-products such as salmon heads (SHs), their brain, eyes and main optic nerves (SBEON), and head-remnants after SBEON removal (RemSH), as well as herring fillets (HFs), herring heads (HHs) and minced boarfish (MB), were evaluated as potential sustainable sources of such bioactive PLs. The antithrombotic bioactivities of PLs derived from these marine by-products were assessed for the first time in human platelets against platelet-activating factor (PAF), thrombin, collagen, and adenosine diphosphate (ADP), while their fatty acid composition was evaluated by gas chromatography–mass spectrometry (GC-MS). PLs from all marine by-products tested possess strong antithrombotic activities against aggregation of human platelets induced by all platelet agonists tested. RemSH, SBEON, HHs, HFs, and MB exhibited strong anti-PAF effects, similar to those previously reported for salmon fillets. PLs from MB had the strongest anti-collagen effects and PLs from SHs and SBEON were the most active against thrombin and ADP. PLs from HHs had similar antithrombotic effects with those from HFs in all agonists. RemSH was less active in all agonists, suggesting that SBEON is the main source of bioactive PLs in SHs. All PLs were rich in omega-3 polyunsaturated fatty acids (ω3PUFA), such as docosahexaenoic acid (DHA) and eicosapentaenoic (EPA) acid, with favourable low values of the ω6/ω3 ratio. Salmon, herring, and boarfish by-products are rich sources of bioactive marine PLs with potent antithrombotic and cardioprotective properties.

## 1. Introduction

Inflammatory and thrombotic events are implicated in all stages of atherosclerosis and cardiovascular disease (CVD) [1,2,3,4,5]. Platelet-activating factor (PAF), thrombin, collagen, adenosine diphosphate (ADP) and their related pathways contribute to activation and aggregation of platelets that play a key role in these manifestations [1,2,3,6,7]. The study of antithrombotic molecules and compounds against these platelet agonists, especially of natural origin such as those derived from food and food by-products, is of potential cardiovascular value [1,8,9].

Indeed, the consumption of fish and fish oils is associated with an improvement of platelet functionality in several disorders [10,11]. These effects were primarily attributed to their high content of ω3 polyunsaturated fatty acids (PUFA), such as eicosapentaenoic acid (EPA) and docosahexaenoic (DHA) acid, and their eicosanoid-related antiplatelet effects [10]. EPA has universally-accepted antiplatelet effects [12], whereas DHA is a structural fatty acid in nervous tissues such as the brain and the retina [8,11,13]. Recent studies have also shown that supplements of EPA at high doses (4 g/day) reduce both major adverse cardiovascular events (MACE) and hard endpoints in the REDUCE-IT clinical trial (Reduction of Cardiovascular Events With Icosapent Ethyl–Intervention Trial) [14], while EPA reduced both first events and recurrent events [15]. Interestingly, the protective effects of ω3 fatty acids are more evident in patients with low fish consumption (as shown in the VITAL clinical trial (Vitamin D and Omega-3 Trial) [16]).

On the other hand, there are several recent reviews and meta-analyses highlighting that marine oil ω3 PUFA supplements (as purified fatty acids or esters and triglycerides) do not affect the risk of major adverse cardiac events, cancer and all-cause death [11,16,17,18,19,20,21,22]. Furthermore, some of these studies suggest that the observed beneficial effects of fish and fish oils are likely to be mediated through the interplay of other beneficial lipid nutrients [11,21,22].

Within this concept, promising outcomes have been attributed to polar lipids (PLs) of marine origin [22], especially those bearing ω3 PUFA within their structure [22,23,24,25]. Such marine PL possess a plethora of beneficial bioactivities against inflammation-related disorders and high bioavailability of their bioactive ω3 PUFA into plasma lipoproteins, cell membranes, and several tissues, including those with difficult accessibility such as the brain [1,8,9,22,23,24,25,26,27]. Marine PLs also possess strong anti-inflammatory, antithrombotic, and cardioprotective activities against platelet-activating factor (PAF)-related pathways and metabolism [1,22,23,24,28], but also against the thrombin pathways [23,24,29,30].

To the best of our knowledge, there is limited but ongoing research on the beneficial effects of PL from several fish species and their by-products [13,22,23,24,31,32,33,34,35,36]. Valuable marine lipid compounds can be recovered from by-products and wastes from the fish industries [13,31,32,33,34,35]. Marine lipids can be extracted from solid residues such as fish heads or minced tissues of very small fish species usually discarded by fisheries, such as boarfish and fish industry processing by-products [13,31,32,33,34,35,36]. The large quantities of by-products generated have great potential for the extraction of biologically desirable and high-value compounds such as marine lipids [13,31,32,33,34,35,36].

In this study, salmon heads (SHs), herring heads (HHs) and herring fillets (HFs), along with minced boarfish (MB) and salmon brain, eyes and main optic nerve (SBEON) and the SH remnants after the removal of the SBEON (RemSH), were evaluated for the first time as effective sources of biofunctional marine PLs with antithrombotic and cardioprotective properties. PL extracts from these marine sources were assessed against aggregation of human platelets induced by the potent inflammatory and thrombotic mediators, namely PAF and thrombin, but also from other well-established platelet agonists such as collagen and ADP. In addition, the fatty acid (FA) composition of the PL extracts from each one of these marine sources was also evaluated for elucidating relationships between structure and observed bioactivities.

## 2. Results

### 2.1. Total, Neutral, and Polar Lipid Content of Salmon, Herring, Boarfish and Their By-Products

The content for total lipids (TLs), neutral lipids (NLs) and PLs is presented in Table 1 and is expressed as g per 100 g of fish tissue for all samples. Salmon, herring, and boarfish are categorised as oily fish species, thus their lipid content is usually higher than other fish species. In the present study too, all salmon, herring, and boarfish by-products had high lipid content as reflected by their TLs, which is in accordance with the literature, apart from the lipid content of HFs for which TL levels were within the range of reported seasonal variations [22,24,27,37,38,39]. Both the SHs and HHs possessed a higher content (*p* < 0.01) of TLs than the previously reported salmon fillets (SFs) [24] and HFs, respectively. MB was found to have similar TL content with that of HHs, which was found to be also similar to previously reported SFs [24], but higher than that of HFs.

In addition, all the TL extracts of these marine sources contain mainly NLs, since the NL fraction contributes approximately to 58%–94% of the TLs, whereas the PL fraction contributes approximately to 6%–42% of the TLs. These results are in accordance with previous studies in these fish species and their related by-products [22,24,36,37,38,39]. No significant difference was observed between the yields of PL extracts in all samples tested, which was generally within the range of approximately 0.4–1.8 g/100 g of tissue, suggesting that all the samples tested seem to be good sources for marine PLs too. Notably, the lipid content of the SBEON is characterized by high levels of PLs, which seems to contribute greatly to the overall PL content of the SHs (approximately 15%–25% of the overall PL in SHs).

### 2.2. Antithrombotic Bioactivities of PLs from Salmon, Herring, Boarfish, and Their By-Products in Human Platelets

**Figure 1:**
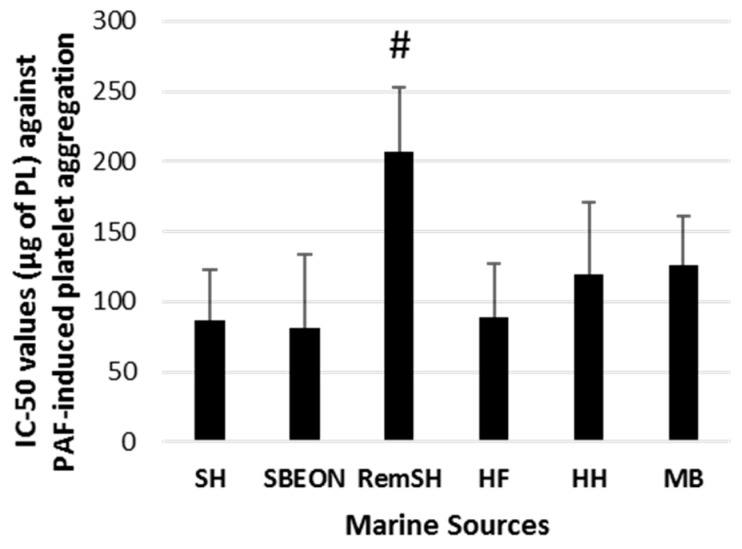
Antithrombotic activities of PL extracts from salmon, herring, and boarfish by-products against platelet-activating factor (PAF)-induced aggregation of human platelets. Results are expressed as IC_50_ (half-maximal inhibitory concentration) values that reflect the inhibitory strength of each PL extract against PAF-induced platelet aggregation and is expressed as mean values of μg of PLs in the aggregometer cuvette that causes 50% of inhibition of PAF-induced aggregation of platelets in human platelet-rich plasma (hPRP) ± SD. It should be noted that the lower the IC_50_ value, the stronger the inhibition against the platelet agonist tested. **^#^** Indicates statistically significant differences of the less bioactive extract (*p* < 0.05). PL: polar lipid; SH: salmon head, SBEON: salmon brain, eyes and main optic nerve; RemSH: the remnants of SH after the removal of SBEON; HF: herring fillet; HH: herring head; MB: minced boarfish; SD: standard deviation.

**Figure 2:**
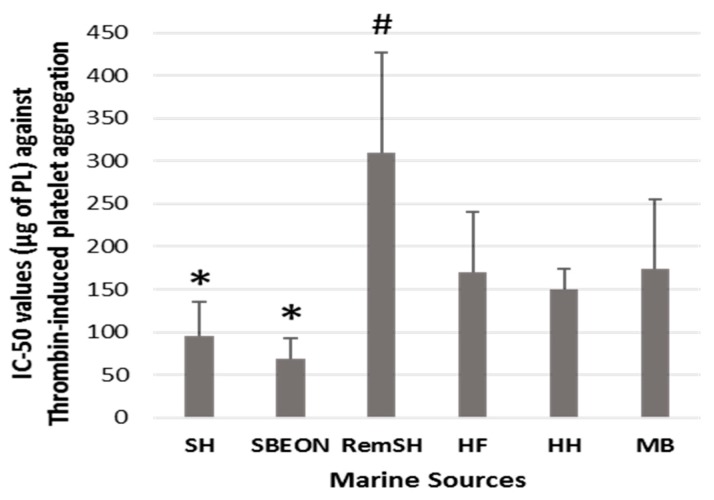
Antithrombotic activities of PL extracts from salmon, herring, and boarfish by-products against thrombin-induced aggregation of human platelets. Results are expressed as IC_50_ (half-maximal inhibitory concentration) values that reflect the inhibitory strength of each PL extract against thrombin-induced platelet aggregation and is expressed as mean values of μg of PLs in the aggregometer cuvette that causes 50% of inhibition of thrombin-induced aggregation of platelets in hPRP ± SD. It should be noted that the lower the IC_50_ value, the stronger the inhibition against the platelet agonist tested. * and **^#^** indicate statistically significant differences (*p* < 0.05) for the most and least bioactive extracts, respectively. PL: polar lipid; SH: salmon head, SBEON: salmon brain, eyes and main optic nerve; RemSH: the remnants of SH after the removal of SBEON; HF: herring fillet; HH: herring head; MB: minced boarfish; hPRP: human platelet-rich plasma; SD: standard deviation.

**Figure 3:**
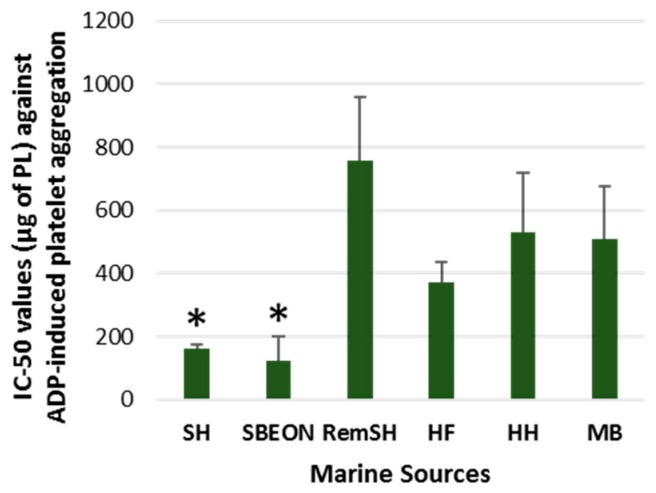
Antithrombotic activities of PL extracts from salmon, herring, and boarfish by-products against adenosine diphosphate (ADP)-induced aggregation of human platelets. Results are expressed as IC_50_ (half-maximal inhibitory concentration) values that reflects the inhibitory strength of each PL extract against ADP-induced platelet aggregation and is expressed as mean values of μg of PLs in the aggregometer cuvette that causes 50% of inhibition of ADP-induced platelet aggregation in hPRP ± SD. It should be noted that the lower the IC_50_ value the stronger the inhibition against the platelet agonist tested. * Indicates statistically significant differences (*p* < 0.05). PL: polar lipid; SH: salmon head, SBEON: salmon brain, eyes and main optic nerve; RemSH: the remnants of SH after the removal of SBEON; HF: herring fillet; HH: herring head; MB: minced boarfish; hPRP: human platelet-rich plasma; SD: standard deviation.

**Figure 4:**
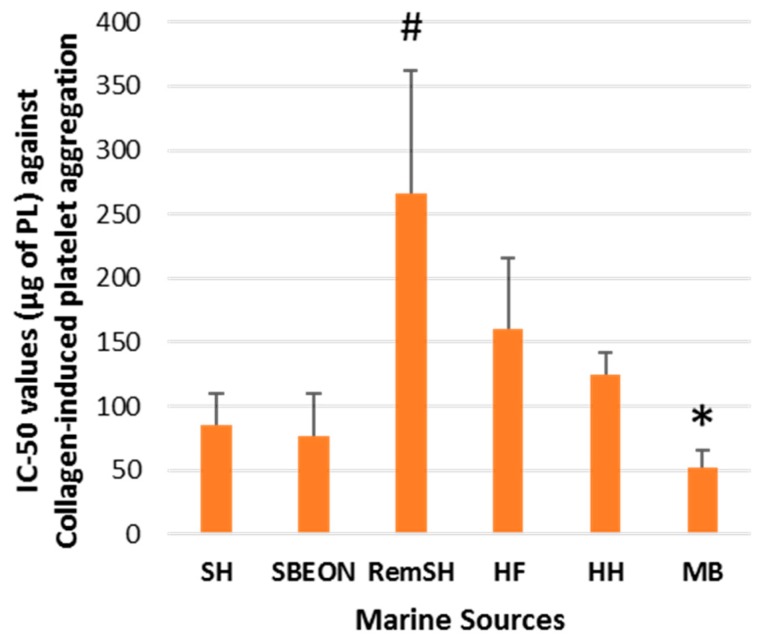
Antithrombotic activities of PL extracts from salmon, herring, and boarfish by-products against collagen-induced aggregation of human platelets. Results are expressed as IC_50_ (half-maximal inhibitory concentration) values that reflect the inhibitory strength of each PL extract against collagen-induced platelet aggregation and is expressed as mean values of μg of PL in the aggregometer cuvette that causes 50% of inhibition of collagen-induced aggregation of platelets in hPRP ± SD. It should be noted that the lower the IC_50_ value, the stronger the inhibition against the platelet agonist tested. * and **^#^** indicate statistically significant differences (*p* < 0.05) for the most and least bioactive extracts, respectively. PL: polar lipid; SH: salmon head, SBEON: salmon brain, eyes and main optic nerve; RemSH: the remnants of SH after the removal of SBEON; HF: herring fillet; HH: herring head; MB: minced boarfish; hPRP: human platelet-rich plasma; SD: standard deviation.

The in vitro antithrombotic activities of PLs from SHs, SBEON, RemSH, HFs, HFs, and MB were evaluated for the first time by their inhibitory effects against human platelet aggregation induced by PAF, thrombin, collagen, and ADP. These effects were expressed by their IC_50_ values (half-maximal inhibitory concentrations) against each platelet agonist. The results for these IC_50_ values are shown in Figure 1, Figure 2, Figure 3 and Figure 4, respectively. It should be noted that the lower the IC_50_ value the stronger the inhibition against the platelet agonist tested.

The IC_50_ values obtained against platelet aggregation, induced by all these platelet agonists, showed that all PL extracts exhibited strong antithrombotic effects. More specifically, apart from the PLs derived from RemSH, PLs from all the other sources (SHs, SBEON, HHs, HFs, and MB) strongly inhibit the PAF pathway of platelet aggregation (Figure 1). These anti-PAF effects were similar (*p* > 0.05) for the PLs from all these marine sources (SHs, SBEON, HHs, HFs, and MB) and with the previously reported anti-PAF effects of the PL extracts of SFs [23,24] *(p* > 0.05), while they were also much stronger than the ones for PL from RemSH (*p* < 0.05) (Figure 1).

Moreover, the PLs from SHs exhibited the strongest inhibitory effects against the thrombin-induced platelet aggregation, in comparison to the PLs from all the other marine sources tested, apart from the PLs from SBEON (Figure 2). The strong anti-thrombin effects of PLs from SHs and SBEON were also significantly higher (*p* < 0.05) than the previously reported anti-thrombin effects for the PL extracts of SFs [24], which were also extracted using the same extraction method [40] and countercurrent distribution method [41].

Furthermore, the PLs from SHs and SBEON exhibited the strongest inhibitory effects against ADP-induced platelet aggregation in comparison to all the other PL extracts tested (*p* < 0.05) (Figure 3), while their strong anti-ADP effects were similar with anti-ADP effects for the PL extracts from SFs.

On the other hand, the PL extracts of MB had the strongest inhibitory effects against collagen-induced platelet aggregation in comparison to the PLs from all the other marine sources tested (*p* < 0.05) (Figure 4), while its strong anti-collagen effects were also significantly higher (*p* < 0.05) when compared with anti-collagen effects of the PLs from SFs (unpublished data). Notably, the fact that the anti-collagen effects of the PL from MB were also significantly stronger than its anti-PAF and anti-thrombin effects and much stronger than its anti-ADP effects (*p* < 0.05 in all comparisons), further suggests that MB seem to contain PL moieties with higher specificity against the collagen pathway.

In contrast to the previously reported results for the PLs from SFs [24], the anti-PAF effects of PLs from SHs were found to be similar with its anti-thrombin and anti-collagen effects (*p* > 0.05), while stronger than its anti-ADP effects (*p* < 0.05), which implies the presence of different bioactive PL moieties in SHs than in SFs. Notably, the PL extracts from SBEON exhibited a similar pattern, since their anti-PAF effects were found to be similar with its anti-thrombin and anti-collagen effects (*p* > 0.05), while stronger than its anti-ADP effects (*p* < 0.05). Furthermore, the high bioactivity of PL extracts from SHs against platelet aggregation induced by all platelet-agonists tested, can be attributed mostly to the PL content of the brain, eyes, and optical nerves. This notion is supported by the fact that the PL extracts of SBEON exhibited similar anti-PAF, anti-thrombin, anti-collagen, and anti-ADP effects when compared to the relative antithrombotic activities of the PLs from SHs (*p* > 0.05), while the PL extracts from RemSH exhibited significantly much lower activities against all these platelet agonists.

In addition, the anti-PAF effects of the PLs from HF were found to be significantly stronger than its anti-thrombin and anti-collagen effects and much stronger than its anti-ADP effects (*p* < 0.05 in all these comparisons), a result that implies a higher specificity of the PL from HF against the PAF pathway. In contrast, the anti-PAF effects of HH were found to be similar to the anti-thrombin and anti-collagen effects (*p* > 0.05), while stronger than its anti-ADP effects (*p* < 0.05), which also implies the presence of different bioactive PL moieties in HF than in HH.

### 2.3. Fatty Acid Composition of PLs from Salmon, Herring, Boarfish, and Their By-Products

The fatty acid composition of PLs from SHs, HHs, HFs, and MB are shown in Table 2. It is clear that these PL samples were rich in PUFA and especially in ω3 PUFA with a very low ratio of ω6/ω3. More specifically, in PLs from SHs, the PUFA were the most abundant fatty acid class followed by the monounsaturated fatty acids (MUFA) and the saturated fatty acids (SFA) (Table 2). PLs from SHs contain high amounts of ω3 PUFA, with the most abundant ω3 fatty acids being DHA (22:6ω3) and EPA (20:5ω3) (Table 2). Considerable but less amounts of ω6 fatty acids were also present with the most abundant being linoleic acid (LA; 18:2ω6) followed by less amounts of arachidonic acid (ARA; 20:4ω6). Furthermore, the most abundant of the MUFA was found to be oleic acid (18:1 c9) and in the SFA, stearic acid (18:0). Interestingly, the overall ω3 fatty acid content of the PLs from SHs was significantly higher than that of ω6 fatty acids and thus the ratio of ω6/ω3 in PLs from SHs was approximately 1/5 (Table 2), which is lower than the value of 1/1 for this ratio.

Similarly, in the HHs PLs the PUFA were the most abundant fatty acid class followed by MUFA and SFA (Table 2). PLs from HHs contain high amounts of ω3 PUFA, with the most abundant ω3 fatty acids being DHA (22:6ω3), while considerable but less amounts of EPA (20:5ω3) were also present (Table 2). Much lower but considerable amounts of ω6 fatty acids were also present with the most abundant being arachidonic acid (ARA; 20:4ω6) and linoleic acid (LA; 18:2ω6). Notably, similar to the PLs from the SHs, the overall ω3 fatty acid content of the PLs from HHs was significantly higher than that of ω6 fatty acids and thus the ratio of ω6/ω3 was found to be approximately 1/10 (Table 2), which is much lower than the value of 1/1 for this ratio. In addition, the most abundant of the MUFA in PL from SHs was found to be oleic acid (18:1 c9) and palmitoleic acid (16:1 c9) and in the SFA the palmitic (16:0) and the stearic acid (18:0).

In contrast, in the PLs from HFs the SFA were the most abundant fatty acid class followed by the PUFA and less amounts of the MUFA (Table 2). Nevertheless, the majority of the PUFA contained in PLs from HFs were found to be ω3 PUFA with the most abundant ω3 fatty acids being DHA (22:6ω3) and EPA (20:5ω3) (Table 2). One to two orders of magnitude, much lower amounts of ω6 PUFAs were contained in PLs from HFs, with the most abundant ω6 fatty acids being arachidonic acid (ARA; 20:4ω6) and linoleic acid (LA; 18:2ω6). Therefore, the overall ω3 fatty acid content of the PL from HFs was significantly higher than that of ω6 fatty acids and thus the ratio of ω6/ω3 was found to be approximately 1/42 (Table 2), which is much lower than the value of 1/1 for this ratio. PLs of HFs contain relatively higher amounts of SFA with the most abundant being palmitic (16:0) followed by less, but considerable, amounts of stearic acid (18:0) and MUFA, with the most abundant of the MUFA being oleic acid (18:1 c9) and docosenoic acid (22:1).

Finally, the SFA were the most abundant fatty acid class in the PL from MB, followed by the MUFA and less amounts of PUFA (Table 2). Nevertheless, the PUFA contained in PLs from MB were found to be mostly ω3 PUFA with the majority of them being this time from EPA (20:5ω3), followed by much less amounts of DHA (22:6ω3) (Table 2). The ω6 PUFA were very low in PLs from MB with the most abundant ω6 fatty acids being arachidonic acid (ARA; 20:4ω6) and linoleic acid (LA; 18:2ω6). Therefore, in PLs from MB the overall ω3 fatty acid content was significantly higher than that of ω6 fatty acids and thus the ratio of ω6/ω3 was found to be approximately lower than 1/53 (Table 2), which is much lower than the value of 1/1 for this ratio. PLs of MB contain relatively higher amounts of SFA with the most abundant being palmitic (16:0) followed by stearic acid (18:0) and myristic acid (14:0), but also considerable amounts of MUFA with the most abundant being oleic acid (18:1 c9) and eicosenoic acids (20:1).

## 3. Discussion

Marine fishery by-products of processing are generated when the fish is gutted, headed, and further processed either on-board fishing vessels or in processing plants on shore. It is estimated that only 50% of total catch is used for actual human consumption [13,31,32,33,34,35,36]. On the other hand, fish heads, viscera, skin, remaining fish muscle proteins, fish bone, tails, offal, and blood possess several biologically valuable and desirable compounds, which can be employed for applications in human health and other industries (i.e., aquaculture, food, agrochemical, biotechnological, and pharmaceutical applications), such as fish oil, collagen, and gelatin production [13,31,32,33,34,35,36].

Lately, the production of bioactive fish oil from such by-products is of great interest, with health benefits that have been attributed to its rich content in ω3 PUFA, such as DHA and EPA [13,31,32,33,34,35,36]. The majority of worldwide fish oil production is mostly used in the aquaculture industry, while only a small proportion is used for the production of ω3 PUFA-related products [33]. Instead, fishing of most species just for the production of oil is not a sensible or sustainable approach, but by using fish waste it is possible to produce considerable amounts of such bioactive fish oil [33]. In addition, such an approach could help to reduce processing waste, thereby catering to ethical and environmental concerns over fish processing discards [13,31,32,33,34,35,36].

Consumption of fish oil rich in ω3 PUFA such as DHA and EPA has been associated with several health benefits, such as improved platelet functionality and cardiovascular health [10,11], while a low value of the ratio of ω6/ω3 PUFA in a diet seems also to provide several beneficial health outcomes in CVD and other chronic disorders [42]. However, recent reviews and meta-analyses have highlighted that marine oil ω3 PUFA supplements such as purified fatty acids, esters or moieties of triglycerides do not effectively affect the risk for chronic disorders as initially thought [11,16,17,18,19,20,21,22], while other beneficial lipid nutrients seem to contribute to the benefits of fish oils [11,21,22].

The therapeutic dose of ω3 fatty acids depends not only on the degree of disease severity, but also on the form that these essential lipids are consumed, with different digestion mechanisms and bioavailability in cell membranes and lipoproteins [11,22,25,26]. Marine DHA or EPA themselves are usually moieties of lipid molecules such as esters, triglycerides (TG), or polar lipids (e.g., phospholipids and glycolipids). Although the most abundant DHA- or EPA-rich lipid class in most marine by-products is TG, some of the fisheries by-product sources or poorly-used marine resources are rich in DHA- or EPA-containing phospholipids that are often called marine phospholipids [36]. TG and esters are typical hydrophobic compounds, while on the other hand, phospholipids are amphiphilic compounds. For this reason, the phospholipid form of DHA and EPA are considered to be much more bioactive and bioavailable than those of TG and esters, but also more effective in delivering the desired PUFA to the desired tissue when administrated, especially in difficult to reach tissues such as the brain because they can surpass the blood–brain barrier [22,23,24,25,35].

Within this concept, promising outcomes have been attributed to PLs of marine origin [22], especially to those bearing ω3 PUFA within their structure [22,23,24,25,26,27], with strong anti-inflammatory, antithrombotic, and cardioprotective activities potentially mediated through anti-PAF and anti-thrombin effects [1,22,23,24,28,29,30].

In the present study, it was found for the first time that several fish and their processing by-products such as the SHs, SBEON, HHs, and MB are effective sources of such bio-functional marine PLs with strong antithrombotic bioactivities against well-established platelet-agonists, namely PAF, thrombin, collagen, and ADP. The strong anti-platelet effects of PL from these marine by-products were also found to be comparable to the relevant effects of the edible parts (fillets) such as HFs and SFs and to other fish species [23,24,43,44,45].

More specifically, PLs from SHs, HHs, and MB were found to strongly inhibit the PAF pathway of human platelet aggregation, an effect that was found to be similar with the PLs from the edible parts (fillets) such as HFs and SFs [23,24], but also with other edible fish species [22,43,44,45]. Moreover, PLs from SHs exhibited a much higher inhibitory effect against the thrombin pathway of human platelet aggregation than previously reported for PLs from SFs [24], which were also extracted by applying the same methodology [40,41], suggesting that SHs seem to possess PL moieties with higher specificity against the thrombin pathway than PL moieties of the edible parts of salmon such as SFs. In contrast to the previously reported results and unpublished data obtained for PL extracts from SFs [24] (unpublished data), the anti-thrombin effects of PL extracts from SHs were similar with its anti-PAF and anti-collagen effects, while stronger than its anti-ADP effects, which further implies the presence of different bioactive PL moieties in SHs than in SFs.

Interestingly, the PLs from SBEON exhibited similar anti-PAF, anti-thrombin, anti-collagen, and anti-ADP effects when compared to relative PLs from SHs, with a similar pattern, while the PLs from RemSH exhibited significantly much lower antithrombotic effects against all these platelet-agonists in human platelets. These results suggest that the strong antithrombotic bioactivities of PL extracts from SHs against platelet aggregation induced by all these platelet agonists can be attributed mostly to its PL content of the brain, eyes, and optic nerves.

Moreover in the present study, the observed potent antithrombotic effects of SHs and SBEON against all these platelet agonists were found to be similar with the ones of the food-grade extracted PLs from SFs [23] (unpublished data), implying that such bioactive SF-derived PL moieties exist in SHs and SBEON also, from where they can be extracted and purified more effectively than from SFs [24] by applying conventional extraction methods [40,41]. However, in order to support the notion that SHs and SBEON possess more effective PL moieties than the SFs against all these platelet agonists, more tests are needed, especially in PLs extracted from SHs and SBEON by applying similar food-grade extraction techniques with those previously reported for obtaining PL from SFs [23] (unpublished data).

Previous elucidation of the structure activity relationship suggested that specific phospholipid moieties bearing ω3 PUFA, such as EPA and DHA, in their *sn*-2 position of their glycerol backbone, either belonging to alkyl-acyl-PLs or acyl-acyl-PLs, seem to be present in most bioactive subclasses of the food grade-extracted PLs from SFs, providing a rational for their strong antithrombotic effects [23,24]. Furthermore, SHs are considered one of the more well-known marine by-products for containing phospholipids beating ω3 PUFA [35,38,39].

In the present study we also found that SHs is a rich source of PLs bearing PUFA within their structure, the majority of which were ω3 PUFA and especially DHA and EPA. It was also found that the ω6/ω3 ratio of the PLs from SHs was lower than the general value of 1/1 for this ratio, which further supports the presence of cardioprotective properties for PLs from SHs since low values for this ratio seem to provide several beneficial health outcomes in CVD and other chronic disorders [42]. Moreover, the content of ω3 PUFA and the observed ω6/ω3 ratio for the PLs from SHs were found to be similar with the previously reported ones for PLs from SFs [24], salmon heads, brain, eyes [38,39], and salmon in general [46,47,48].

On the other hand, the overall content of PUFA in the PLs of HFs, including the total amount of ω3 PUFA and that of DHA and EPA, was found to be lower than their contents in SFA and MUFA. These results on the overall fatty acid composition of PLs from HFs comes in accordance with previously reported ones for the TLs of several species of herring [37,49,50,51].

In contrast to the PLs from HFs, the PLs from HHs were found to have a higher content of PUFA within their structure than that of MUFA and SFA, with ω3 PUFA and especially DHA and EPA being the most abundant ones. Thus, similarly to the SH by-product from salmon, the HH by-product from herring seems also to be a rich source of PLs bearing ω3 PUFA, a result that comes in accordance with previous reported ones for HHs [50]. Notably, the ω6/ω3 ratio for both the PLs from HHs and HFs was at least one order of magnitude lower than the general value of 1/1 for this ratio. This result further supports the beneficial properties for the PLs of either the fillet from herring or its head by-product too, since the lower the value for this ratio the better the health outcomes in the aforementioned chronic disorders [42].

However, differences observed in the antithrombotic specificity of PLs from HFs against each platelet agonist were not similar with the ones observed in PLs from HHs, implying the presence of different bioactive PL moieties in HFs than in HHs. More specifically, the PLs from HFs had stronger anti-PAF effects than their anti-thrombin, anti-collagen, and anti-ADP effects, suggesting a higher specificity against the PAF pathway for the PLs from HFs. In contrast, in the PLs from HHs the anti-PAF effects were similar with their anti-thrombin and anti-collagen effects, while stronger than their anti-ADP effects. Nevertheless, the overall strong antithrombotic effects of the PLs from HHs were similar with those of the PLs from HFs.

Notably, from all the samples tested, the PLs from MB possessed the strongest inhibitory effects against the collagen-induced platelet aggregation, an anti-collagen effect that was stronger even from the effects of PL extracts from SFs (unpublished data). These results, in combination with the fact that the anti-collagen effects of the PL extracts of MB were also significantly stronger than its anti-PAF and anti-thrombin effects, and much stronger than its anti-ADP effects, further support the notion that MB contains PL moieties with higher specificity against the collagen pathway. In addition, in the PLs from MB, even though their content in PUFA were lower than those in SFA and MUFA, these PUFA were mostly ω3 PUFA, with the majority being EPA and less being DHA. Therefore, the overall ω3 fatty acid content in the PLs from MB was significantly higher than that of their ω6 fatty acids and thus the ratio of ω6/ω3 was found to be the lowest from all the samples tested and much lower than the value of 1/1 for this ratio, supporting the presence of cardioprotective properties for PLs from MB too [42].

Overall, our novel results exhibited that it is worth pursuing the valorisation of several marine by-products as potential sustainable sources of bioactive marine PL with strong antithrombotic and cardioprotective properties against platelet aggregation. Nevertheless, more studies are needed to further support this perspective, such as studies related to direct thrombus formation (for example by using the Badimon chamber), in order to evaluate both platelet aggregation and coagulation [52]. Indeed, it is a limitation of the study that not all platelet agonists were assessed e.g., ARA and epinephrine. Furthermore, clinical trials are required to confirm the presence of these bioactivities in vivo.

## 4. Materials and Methods

### 4.1. Materials and Instrumentation

All glass and plastic consumables, reagents, and solvents were of analytical grade and were purchased from Fisher Scientific Ltd. (Dublin, Ireland), and 20 G safety needles and evacuated sodium citrate S-monovettes for blood sampling were purchased from Sarstedt Ltd. (Wexford, Ireland). The platelet aggregation bioassay was carried out on a Chronolog-490 two channel turbidimetric platelet aggregometer (Havertown, PA, USA), coupled to the accompanying AGGRO/LINK software package. All platelet aggregation consumables were purchased from Labmedics LLP (Abingdon on Thames, UK). Standard PAF, thrombin, and Bovine Serum Albumin (BSA) were purchased from Sigma Aldrich (Wicklow, Ireland), while collagen and ADP were from CHRONOLOG (Havertown, PA, USA). Centrifugations were carried out on an Eppendorf 5702R centrifuge (Eppendorf Ltd., Stevenage, UK). Spectrophotometric analysis was carried out on a Shimadzu UV-1800 spectrophotometer (Kyoto, Japan).

### 4.2. Samples of Salmon, Herring, Boarfish, and Their By-Products Assessed

Sustainable fish sources and their processing by-products were chosen for this study; samples of salmon heads (*n* = 6) from Irish organic farmed salmon (Salmo salar) were donated by Marine Harvest Ltd. (Donegal, Ireland), herring samples (*n* = 6) were purchased from the local market, while samples of minced boarfish (*n* = 3) were kindly donated by Aurélien V. Le Gouic and Richard J. FitzGerald of the Department of Biological Sciences of the University of Limerick

### 4.3. Extraction and Isolation of Total Lipid, Neutral Lipid, and Polar Lipid Extracts from Salmon, Herring, Boarfish, and Their By-Products

Several samples of SH, SBEON, RemSH, HH, HF, and MB were homogenised mechanically using a Waring blender (Fisher Scientific Ltd., Dublin, Ireland) and their TLs were extracted as previously described [24] using the Bligh and Dyer extraction method [40]. TL extracts of all these marine sources were further separated into their NL and PL fractions as previously described [24], based on the countercurrent distribution method of Galanos and Kapoulas [41].

Solvents were evaporated from all extracts using flash rotary evaporation at a maximum of 40 °C (Buchi Rotavapor, Mason Technology Ltd., Dublin, Ireland) and lipid samples were transferred into small glass vials, where all the remaining solvents were further evaporated under a stream of nitrogen. The acquired TL, NL, and PL extracts were weighed and stored under a nitrogen atmosphere at −20 °C for further analysis

### 4.4. Human Platelet Aggregation Studies of PL Extracts from Salmon, Herring, Boarfish, and Their By-Products against PAF, Thrombin, Collagen, and ADP

Blood collection from several healthy donors (*n* = 12) and preparation of human platelet-rich plasma (hPRP) was conducted as previously described [23,24,53]. The Ethics Committee of the University of Limerick approved the protocol, which was performed in accordance with the Declaration of Helsinki. Healthy donors were fully aware that their blood samples were used in our study and written consent was provided.

Briefly, the blood samples were collected from each donor by a specialised phlebotomist using sodium citrate anticoagulant (0.106 mol/L in a 1:10 ratio of citrate to blood; Sarstedt Ltd., Wexford, Ireland) and were centrifuged at 194× *g* for 18 minutes at 24 °C with no brake applied. The supernatant hPRP was then transferred to polypropylene tubes at room temperature for the aggregation bioassays, whereas platelet-poor plasma (PPP) was obtained by further centrifuging the remainder of the blood specimens at 1465× *g* for 20 minutes at 24 °C with no brake applied. hPRP was adjusted to 500,000 platelets/µL if required by addition of the respective volume of PPP according to the absorbance of the hPRP measured using a spectrophotometer at 530 nm (Shimadzu UV-1800, Kyoto, Japan).

Aliquots of standard PAF stock solution in chloroform/methanol (1:1 *v*/*v*) were evaporated under a stream of nitrogen and re-dissolved in BSA (2.5 mg BSA/mL saline) to obtain PAF solutions with final concentrations in the aggregometer cuvette ranging from 0.26 nM to 0.26 μM. The examined salmon PL samples were also dissolved in BSA (2.5 mg BSA/mL saline). Standard stock solutions of active thrombin, collagen, and ADP dissolved in saline were further diluted in saline prior testing.

Then, 250 µL of PRP was added to an aggregometer cuvette at 37 °C with stirring at 1000 rpm. The PRP was calibrated using the PPP as a blank. The maximum-reversible platelet aggregation curve induced by PAF, thrombin, collagen or ADP was determined as 100% aggregation, which was also used as a baseline (0% inhibition) in the absence of any lipid sample, by adding the appropriate amounts of each platelet agonist in the aggregometer cuvette, in order to reach specific final concentrations: for PAF approximately 0.1–1 nM, for thrombin approximately 0.01–0.4 U/mL, for collagen approximately 1–5 μg/mL and for ADP at approximately 2–10 μΜ.

The PAF, thrombin, collagen, and ADP-induced aggregation of hPRP was calculated first at 0% inhibition of baseline in a cuvette (100% aggregation) in the absence of any lipid sample, whereas after the pre-incubation of hPRP with several amounts (μg) of the lipid samples in a different cuvette for 2 min, the same amount of PAF, thrombin, collagen, or ADP that induced maximum-reversible platelet aggregation was added and the reduced aggregation was calculated. Thus, a linear curve at the 20%–80% range of the percentage of inhibition against PAF, thrombin, collagen, and ADP-induced aggregation of hPRP was deduced for each sample. From this curve, the concentration (μg) of the lipid sample that led to 50% of agonist-induced aggregation of hPRP was calculated as the 50% inhibitory concentration value also known as the IC_50_ value (half-maximal inhibitory concentration) for each sample. The resulting IC_50_ values were expressed as a mean value of the mass of lipid (µg) in the aggregometer cuvette ± standard deviation (SD). All experiments were performed several times (*n* ≥ 10), using a different donors’ blood samples for each replicate to ensure reproducibility.

### 4.5. Gas Chromatography–Mass Spectrometry of PL Extracts from Salmon, Herring, Boarfish, and Their By-Products

GC-MS analysis of the FA composition of PL extracts from SHs, HHs, HFs, and MB was carried out as previously described [24].

### 4.6. Statistical Analysis

One-way analysis of variance (ANOVA) was used in comparisons of all IC_50_ values against PAF, thrombin, collagen, and ADP platelet-aggregation and with previously reported ones for SFs against all these agonists [23,24] (unpublished data), while Kruskal–Wallis non-parametric multiple comparison test was used for comparisons in the FA composition acquired from the GC-MS analysis. Differences were considered to be statistically significant when the *p*-value was less than 0.05 (*p* < 0.05). The Kolmogorov–Smirnov criterion was used for normality in the distribution of the values for all variables. The data were analysed using a statistical software package (IBM-SPSS statistics 25 for Windows, SPSS Inc., Chicago, IL, USA).

## 5. Conclusions

In the present study it was found for the first time that several fish (salmon, herring, and boarfish) and their processing by-products (SHs, SBEON, HHs, and MB) contain bioactive PLs with strong antithrombotic effects against human platelet aggregation induced by well-established potent platelet agonists, namely PAF, thrombin, collagen, and ADP. Our novel results support the valorisation of such marine by-products as sources of bioactive marine PLs with antithrombotic bio-functionality and cardioprotective properties that may facilitate a sustainable design of novel food supplements and nutraceuticals against platelet and inflammation-related disorders such as CVD. However, further studies are required to further support this notion.

## Figures and Tables

**Table 1:** Lipid content (TLs, PLs, and NLs) of SH, SBEON, RemSH, HH, HF, and MB.

Marine Source	TLs *	NLs *	PLs *
**SH**	15.3 ± 5.3 ^#^	14.2 ± 4.4	1.1 ± 0.1
**SBEON**	1.2 ± 0.4	1.0 ± 0.1	0.2 ± 0.1
**RemSH**	13.9 ± 2.3	13 ± 2.1	0.9 ± 0.2
**HF**	1.2 ± 0.3	0.7 ± 0.2	0.4 ± 0.1
**HH**	8.6 ± 0.7^#^	6.2 ± 0.4	0.9 ± 0.1
**MB**	7.3 ± 0.2	5.9 ± 0.2	1.2 ± 0.2

* Expressed as mean values of g of lipids per 100 g of tissue from each marine source (mean ± SD, *n* = 6); TLs: total lipids; PLs: polar lipids; NLs: neutral lipids; SH: salmon head; SBEON: salmon brain, eyes and main optic nerve; RemSH: the remnants of SH after the removal of SBEON; HF: herring fillet; HH: herring head; MB: minced boarfish; SD: standard deviation. ^#^ Indicates statistically significant difference (*p* < 0.05) of the TL content of heads when compared with the relative TL content of fillets from the same fish species; the results for SH were compared with previously reported ones by Tsoupras et al. [19] for salmon fillet (SF).

**Table 2:** The fatty acid profile of the polar lipid extracts of herring fillets and heads, salmon heads and minced boarfish expressed as a percentage of the total fatty acids of each sample (mean ± SD, *n* = 3).

Fatty Acid	HF	HH	SH	MB
**10:0**	ND	ND	ND	0.015 ± 0.002 ^a^
**12:0**	ND	ND	0.001 ± 0.000 ^a^	0.062 ± 0.003 ^b^
**14:0**	0.477 ± 0.015 ^a^	3.101 ± 0.364 ^b^	1.401 ± 0.110 ^a^	12.76 ± 0.798 ^c^
**13-me-14:0**	0.024 ± 0.008 ^a^	0.013 ± 0.001 ^a^	ND	ND
**14:1**	ND	0.015 ± 0.002 ^a^	0.019 ± 0.001 ^a^	0.177 ± 0.020 ^b^
**15:0**	0.079 ± 0.017 ^ab^	0.544 ± 0.096 ^c^	0.019 ± 0.000 ^a^	0.160 ± 0.018 ^b^
**16:0**	48.74 ± 2.352 ^a^	19.89 ± 0.314 ^b^	1.367 ± 0.071 ^a^	32.17 ± 1.565 ^c^
**15-me-16:0**	0.012 ± 0.003 ^a^	0.035 ± 0.022 ^a^	0.022 ± 0.002 ^a^	0.015 ± 0.005 ^b^
**16:1 c9**	0.330 ± 0.044 ^a^	5.686 ± 0.531 ^e^	1.854 ± 0.045 ^c^	1.340 ± 0.085 ^bc^
**16:1 c6**	ND	0.992 ± 0.052	ND	ND
**17:0**	0.151 ± 0.006 ^ab^	0.325 ± 0.022 ^b^	0.078 ± 0.007 ^ab^	0.054 ± 0.008 ^ab^
**17:1**	0.016 ± 0.005 ^a^	0.044 ± 0.004 ^c^	ND	0.089 ± 0.012 ^d^
**18:0**	8.621 ± 0.144 ^c^	5.088 ± 0.063 ^a^	5.732 ± 0.170 ^b^	15.39 ± 0.281 ^e^
**18:1 c9**	8.969 ± 0.576 ^a^	13.30 ± 0.381 ^b^	14.16 ± 1.341 ^bc^	13.63 ± 0.337 ^b^
**18:1 c11**	ND	0.410 ± 0.040 ^a^	2.388 ± 0.229 ^b^	ND
**18:1 t13**	0.267 ± 0.012 ^c^	0.045 ± 0.002 ^ab^	0.029 ± 0.006 ^a^	0.253 ± 0.007 ^c^
**18:2 c9, c12**	0.242 ± 0.012 ^a^	1.205 ± 0.063 ^b^	8.822 ± 0.639 ^c^	0.088 ± 0.007 ^a^
**18:2 c9, t11**	0.064 ± 0.017 ^a^	0.707 ± 0.114 ^b^	0.039 ± 0.004 ^a^	ND
**18:3 c9, c12, c15**	0.269 ± 0.028 ^ab^	0.538 ± 0.031 ^b^	1.496 ± 0.289 ^c^	0.026 ± 0.005 ^a^
**20:0**	ND	ND	0.028 ± 0.007	ND
**20:1 c11 or c13**	0.217 ± 0.036 ^a^	0.0296 ± 0.001 ^a^	3.370 ± 0.471 ^b^	11.61 ± 0.760 ^c^
**20:4 c5, c8, c11, c14**	0.343 ± 0.051	1.796 ± 0.206 ^b^	2.404 ± 0.147 ^c^	0.160 ± 0.008 ^a^
**20:5 c5, c8, c11, c14, c17**	10.12 ± 0.642 ^d^	8.776 ± 0.505 ^c^	10.89 ± 0.494 ^d^	12.26 ± 0.761 ^e^
**22:0**	ND	ND	0.708 ± 0.564	ND
**22:1**	4.962 ± 0.830 ^c^	2.994 ± 0.277 ^c^	2.681 ± 0.663 ^b^	0.159 ± 0.008 ^a^
**22:5**	0.063 ± 0.007 ^a^	1.751 ± 0.480 ^b^	4.010 ± 0.289 ^c^	ND
**22:6**	16.79 ± 0.911 ^c^	25.62 ± 0.142 ^d^	36.87 ± 0.464 ^e^	0.501 ± 0.058 ^a^
**ω3**	27.25 ± 1.308	36.39 ± 0.768	53.27 ± 1.441	12.79 ± 0.447
**ω6**	0.650 ± 0.034	3.707 ± 0.198	11.26 ± 0.651	0.248 ± 0.098
**ω6/ω3**	0.024 ± 0.002	0.101 ± 0.007	0.211 ± 0.018	0.019 ± 0.008
**SFA**	58.07 ± 2.491 ^de^	28.95 ± 0.756 ^b^	9.588 ± 0.577 ^a^	60.60 ± 2.105 ^e^
**MUFA**	14.76 ± 0.428 ^a^	29.52 ± 0.601 ^de^	24.51 ± 2.370 ^bc^	27.10 ± 0.990 ^cd^
**PUFA**	27.90 ± 1.303 ^b^	40.40 ± 0.958 ^c^	60.53 ± 1.866 ^d^	12.78 ± 0.451 ^a^

^a,b,c^ Mean values (*n* = 3), ± standard deviation with different letters in the same row indicating statistically significant differences between the lipid compositions when means are compared using Kruskal–Wallis non-parametric multiple comparison test (*p* ≤ 0.05 for all ^a,b,c,d,e^ comparisons). Abbreviation: HF: herring fillet; HH: herring head; SH: salmon head; MB: minced boarfish; c: cis; t: trans; me: methyl; SFA: saturated fatty acids; MUFA: monounsaturated fatty acids; ω3: omega-3 PUFA; ω6: omega-6 PUFA; PUFA: polyunsaturated fatty acids; ND: non-detectable.
